# Online Singing Groups for People With Dementia: Adaptation and Resilience in the Face of the COVID-19 Pandemic

**DOI:** 10.1177/14713012231179262

**Published:** 2023-06-09

**Authors:** Becky Dowson, Justine Schneider, Orii McDermott, Martin Orrell

**Affiliations:** Institute of Mental Health, 6123 University of Nottingham, Nottingham, UK; School of Sociology and Social Policy, 6123 University of Nottingham, Nottingham, UK; Institute of Mental Health, 6123 University of Nottingham, Nottingham, UK; School of Sociology and Social Policy, 6123 University of Nottingham, Nottingham, UK; Institute of Mental Health, 6123 University of Nottingham, Nottingham, UK; School of Medicine, 6123 University of Nottingham, Nottingham, UK; Institute of Mental Health, 6123 University of Nottingham, Nottingham, UK; School of Medicine, 6123 University of Nottingham, Nottingham, UK

**Keywords:** Dementia, Alzheimer’s, carers, singing, music, music therapy, arts and health, technology, internet, video calling, COVID-19, coronavirus, telehealth

## Abstract

**Introduction:**

At the start of the COVID-19 pandemic, people with dementia living in the community experienced the sudden loss of their usual activities, and videoconferencing was widely adopted by music groups whilst face-to-face sessions were not possible. This paper reports the findings of a proof-of-concept study of online singing for people living with dementia and their carers, focusing on the experiences of the participants.

**Method:**

People with dementia and their care partners were invited to take part in 10 weeks of online singing sessions. Each session lasted 1 hour, and comprised time for talking, warming up and singing familiar songs. Participants completed standardised outcome measures at baseline and after 10 weeks. Dyads were invited to take part in a semi-structured interview.

**Results:**

In total, 16 pairs were recruited. The response to the online singing group was mostly positive. Participants were able to use the technology to join the sessions, and reported few technical problems. Despite the limitations of online singing, the experience was frequently reported to be enjoyable. Some participants described longer-term benefits, such as improved mood and better relationship between care partners. Some felt online sessions had advantages over face-to-face ones; for example, they were more accessible. However, participants who had previously been attending face-to-face sessions felt that the online singing was a “better than nothing” substitute.

**Conclusions:**

Online singing cannot recreate the experience of group singing face-to-face, and it requires some technical knowledge, but it provides a worthwhile alternative in a time of need for some people with dementia and their carers. Furthermore, for some people online singing may be preferable due to its accessibility. Given the potential for online singing to include people who cannot go out for any reason and its relatively low cost, providers may wish to consider hybrid online/in-person singing groups in future.

## Introduction

People with dementia and their carers have faced threats to their wellbeing since the start of the COVID-19 pandemic. They have been unable to access their usual activities and forms of support, leading to a loss of contact and routine, and they have an elevated risk of serious illness from the virus (Brooke & Jackson, 2020; Tousi, 2020). A review concluded that “the COVID-19 pandemic has had a disproportionately negative impact on people affected by dementia” (Liu et al., 2021: p. 1598). Qualitative evidence from the UK points to increased anxiety and irritability among people with dementia, with some reports that the lockdown restrictions accelerated cognitive decline (Tuijt et al., 2021). People with dementia keenly felt the loss of their routine social activities, like community groups and day centres, as well as loss of contact with their friends. Carers faced sudden restructuring and reduction of formal and casual support, with some changing their living arrangements at short notice to support relatives with dementia.

One factor which was reported to positively affect resilience was the ability to adapt to use technology to maintain contact and sustain hobbies (Hanna et al., 2021). Many forms of face-to-face support for people with dementia and their carers switched to online delivery. Access to online activities relies on people with dementia and carers having the necessary technology, as well as the confidence to use it independently. There is a risk of digital exclusion of people who do not have access to internet-enabled devices and a reliable connection.

Singing groups are a popular activity which have seen widespread adoption by people with dementia and their carers, and the potential benefits include: improved cognitive function and reduced depression (Sarkamo et al., 2016); improved memory, mood and social inclusion (Osman, Tischler, & Schneider, 2014); strengthening of the relationship within caring dyads (Tamplin et al., 2018; Unadkat et al., 2016); and simply providing an enjoyable and worthwhile activity (Camic, Williams, & Meeten, 2011). Musical activities for people with dementia are among those which made the change to digital delivery (Dowson, Atkinson, et al., 2021). A study of facilitators delivering online singing groups for people with dementia in Ireland recognises resilience and adaptability in those running the groups and those attending them, but also highlights the risk of digital exclusion (Lee et al., 2021). However, the novelty of the online delivery mode means that to the best of our knowledge there is no published research directly reporting the experiences of people with dementia and carers who attend these groups. There are certain features of the face-to-face singing experience which may suffer when they are transferred to an online format, and these may affect the success of the session. This study aimed to provide proof-of-concept for online singing, and to explore its implications for the future through understanding the motivations and experiences of the participants.

The transition from face-to-face to online delivery of singing groups is mirrored in the experience of the authors of this paper. In 2020 we were beginning to recruit for a randomised controlled trial of community singing groups for people living with dementia (Dowson, Schneider, et al., 2021). Disruption caused by the pandemic resulted in the suspension of that study, and its eventual adaptation into the work which is reported in this article.

## Aims and Research Questions

The study aimed to evaluate aspects of the online singing process, including technical challenges and participants’ experiences. We hypothesised that the active ingredients of online singing would be broadly the same as for face-to-face singing groups and that its outcomes would be similar. We set out to explore the motivations of the participants for attending the group singing online, including a snapshot of how their lives had changed due to the pandemic, their experiences of the online group, barriers and facilitators to their attendance, advantages and drawbacks of the online group and any benefits they experienced from attending.

## Methods

### Study Design

The study was designed to provide proof of concept for online singing. We recruited an opportunistic sample of people with dementia and their carers who were invited to attend the online singing group weekly for 10 weeks. We carried out semi-structured, post-intervention interviews with participants. We kept records of recruitment, retention and attendance, and monitored the facilitators’ adherence to the intervention protocol. We also collected quantitative data in the form of questionnaires.

### Ethical Approval

This study was reviewed by the Health and Social Care Research Ethics Committee in the United Kingdom and received a favourable ethical opinion (reference 19/IEC08/0056).

### Inclusion and exclusion Criterion

Participants residing in England with a diagnosis of mild or moderate dementia were included if they had a carer who spent at least 2 hours per week with them, could speak and understand English, and had capacity to give informed consent at the start of the study. They were excluded if they had a significant hearing impairment, a severe mental illness or drug/alcohol addiction, or if they were already participating in an interventional study. Carers residing in England were included if they could speak and understand English and were able to give informed consent.

Participants also needed to be able to access Zoom videoconferencing in order to take part in the study. We were able to provide technical support for anyone who was uncertain about using the technology but were unable to provide the hardware devices.

### Sample Size

The recruitment target for the study was 20 dyads, each comprising one person with dementia and their carer. The sample size was chosen pragmatically based partly upon the maximum feasible size for an online singing group and time available.

### Recruitment

Recruitment was undertaken using adverts and posts placed on social media, and by sharing the study advert with relevant organisations and networks. We also used the online register “Join Dementia Research” to aid recruitment; potential participants were contacted directly.

### Informed Consent

Potential participants were initially sent information sheets by email. A video-call with the researcher was then organised, to explain what participation would involve and answer questions. The dyad was then sent a link to an online consent form using Microsoft Forms to confirm participation.

### Intervention

An online singing group was delivered using the videoconferencing platform Zoom. Participants in the study were invited to attend a weekly session for 10 weeks. They either attended an existing online singing session, or one set up for the study. Which session they attended was decided by (1) at what point in the study they signed up and (2) their preference for morning or afternoon sessions. Sessions usually had around 15–20 participants. The sessions were led by two professional community musicians with extensive experience of running face-to-face singing sessions for people with dementia. The two musicians were physically present together for their delivery of the sessions, with one facilitator generally leading the session and the singing, while the other accompanied on guitar (under restrictions at the time, it was permissible for the musicians to be in the same space for the purposes of work, and social distancing and ventilation precautions were taken). The sessions lasted approximately 1 hour and were sometimes slightly longer (for example, if an extra 5 minutes was needed so that everybody had the chance to choose a song).

The day before each session, the facilitator sent out an email containing the details for joining the session. The facilitator started the session a few minutes early and after giving the participants time to join, welcomed everyone to the group. There was then a period of 15–20 min during which the facilitator greeted each dyad and discussed the theme of the week. Themes were based on seasonal events, relevant links to songs, or non-intrusive personal trivia, such as ‘what is your favourite colour?’.

The facilitator then led the participants in a physical and vocal warm up, using gentle exercises to prepare bodies and voices for singing. The warm up usually lasted 10–15 min, after which the group sang from a selection of songs compiled for the study. Songbooks were provided and lyrics were shared on the screen. Each dyad was invited by the facilitator to choose a song most weeks. A list of songs contained in the songbook is provided as supplementary material.

Singing group participants remained muted throughout the session, including for the singing itself. If participants had been unmuted while singing along with the facilitator the overall result would have sounded confusing and asynchronous, due to the audio latency inherent in video calling. For the spoken parts of the session, it was preferable if participants were muted when they were not speaking themselves to minimise background noise and disruption. Participants were shown how to unmute themselves at any time when they wished to speak. Participant cameras were switched on by default, but they could be turned off according to personal preference.

### Data Collection and Analysis

Quantitative measures were used to collect data from participants before the start of the singing group and after 10 weeks of sessions. The data collection with the people with dementia took place via video call. The carers’ data collection was conducted using an online form. Table 1 below outlines the measures which were used and how they were administered. The baseline measures were administered in the 2 weeks prior to the dyad starting their singing sessions, and the post-intervention measures were administered in the 2 weeks following their conclusion. Due to the exploratory nature of the study and the absence of a control condition, the quantitative data was subjected to descriptive analysis only and no statistical testing was used.

**Table 1.** Quantitative measures and methods of administration.

Name and description of instrument		Participant completing instrument		Principal method of administration*	
		Person with dementia	Carer	Video call	Online form
**QoL-AD** (Logsdon et al., 1999)	Quality of life	X		X	
**EQ-5D-5 L** (Kunz, 2010)	Health-related quality of life	X	X	X	X
**DEMQOL** (Rowen et al., 2012)	Quality of life	X		X	
**GDS** (Yesavage et al., 1982)	Depression	X	X	X	X
**MuseEQ** (Vanstone et al., 2016)	Musical engagement	X		X	
**QoL-AD proxy** (Logsdon et al., 1999)	Quality of life		X		X
**IQCODE** (Jorm et al., 1989)	Cognitive decline		X		X
**BADLS** (Bucks et al., 1996)	Activities of daily living		X		X
**QCPR** (Spruytte et al., 2002)	Caring relationship		X		X
**COPE** (McKee et al., 2003)	Experiences/consequences of caring		X		X
**SSCQ** (Vernooij-Dassen et al., 1999)	Experiences of caring		X		X

* Where a measure is marked as being administered both by video call and online form, this means that the person with dementia completed it by video call and the carer by online form.

The interviews were conducted online and dyads were interviewed together for 20–30 min. The interviews were recorded and the recordings were transcribed using the University of Nottingham’s secure automated transcription service, then reviewed and edited for accuracy by BD. Transcripts were analysed in NViVo using the general inductive approach (Thomas, 2006), meaning that the analysis was guided by the evaluation objectives. The initial analysis and coding were completed by BD and then reviewed by JS who suggested changes and refinements. Subsequent iterations of the coding were reviewed and agreed by both researchers.

## Findings

**Figure 1.**
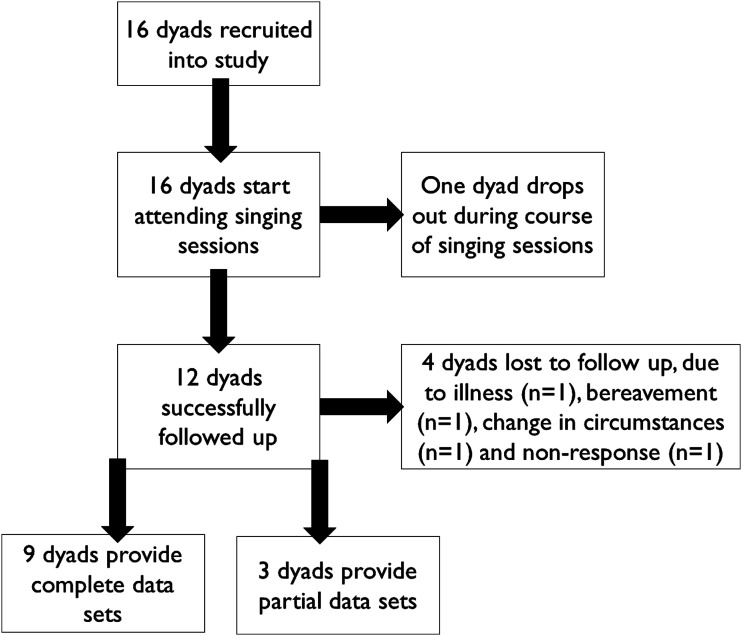
CONSORT diagram of participant flow through the study.

Recruitment commenced in January 2021 and closed in June 2021, enrolling 16 participants with dementia and their carers into the study (80% of the target number). The first participants began attending a singing group in January 2021, and the final participants completed their course of sessions in August 2021. Figure 1 shows the trajectory of participants through the study. Three participants were resident in the same care home and attended the sessions together, and one staff member acted as carer participant for all of them, so the total number of unique carers was 14. The total number of participants in the study including both people with dementia and carers was 30.

**Table 2.** Demographic and background information for participants.

		**Participants with dementia**	**Carers**
**Sex**	Male	7 (43.8%)	2 (14.3%)
	Female	9 (56.3%)	12 (85.7%)
**Age**		72–92 years (mean = 81.06, median = 81.50, S.D. = 6.95)	47–76 (mean = 63.3, median = 62.0, S.D. = 9.63)
**Ethnicity**	White	13 (81.3%)	13 (92.9%)
	Asian	1 (6.3%)	1 (7.1%)
	Other	1 (6.3%)	0
	Not stated	1 (6.3%)	0
**Highest level of education attended**	Up to GCSEs/O Levels or equivalent	5 (31.3%)	0
	A levels or equivalent	4 (25%)	3 (21.4%)
	Undergraduate degree	4 (25%)	7 (50.0%)
	Postgraduate degree	2 (12.5%)	4 (28.6%)
	Not stated	1 (6.3%)	0
**Dementia diagnosis**	Alzheimer’s disease	6 (37.5%)	
	Vascular dementia	3 (18.8%)	
	Mixed (vascular and Alzheimer’s)	2 (12.5%)	
	Frontotemporal dementia	1 (6.3%)	
	Not known	2 (12.5%)	
**Time since dementia diagnosis**		0.25–8.67 years (mean = 4.42, median = 4.43, SD = 2.96)	
**Singing experience**	None at all	1 (6.3)	4 (28.6%)
	Not much	3 (18.8%)	3 (21.4%)
	Quite a lot	6 (37.5%)	5 (35.7%)
	Extensive	5 (31.3%)	2 (14.3%)
	Not stated	1 (6.3%)	0
**Relationship to person with dementia**	Child		7 (50.0%)
	Spouse/Partner		5 (35.7%)
	Friend		1 (7.1%)
	Professional carer		1 (7.1%)

Table 2 summarises the demographic and background information for the study participants.

Table 3 shows which participants took part in data collection at each time point.

**Table 3.** Data collection by dyad.

Dyad number	Questionnaires - baseline		Questionnaires – follow up		Interview	
	Carer	Person with dementia	Carer	Person with dementia	Carer	Person with dementia
1	✓	✓	✓	✓	✓	✓
2	✓	✓	✗^c^	✗^c^	✗^c^	✗^c^
3	✓	✓	✗^c^	✗^c^	✗^c^	✗^c^
4	✓	✓	✓	✓	✓	✓
5	✓	✓	✓	✓	✓	✓
6	✓	✓	✓	✓	✓	✓
7	✓	✓	✓	✓	✓	✗
8	✗^a^	✗^a^	✗^a^	✗^a^	✓	✗
9	✗^a^	✗^a^	✗^a^	✗^a^	✓	✓
10	✓	✓	✗^c^	✗^c^	✗^c^	✗^c^
11	✓	✓	✓	✓	✓	✓
12	✓	✓	✗^b^	✗^b^	✗^b^	✗^b^
13	✓	✓	✓	✓	✓	✓
14	✓	✗^b^	✓	✗^b^	✓	✗^b^
15	✓	✓	✓	✗^b^	✓	✗^b^
16	✓	✓	✓	✗^b^	✓	✗^b^

^a^ = did not participate in quant. data collection as already attending an online singing group.

^b^ = responded to request for interview/questionnaire completion but declined to take part.

^c^ = did not respond to request i.e. lost to follow up.

### Singing group attendance

Participants joined either an existing online singing group, or one which was created for the study. Attendance at the group reached a mean of 79% across the course of 10 sessions. No dyads officially dropped out of the study, but two dyads had sporadic attendance throughout the study and were in the end lost to follow up. The online singing sessions were able to continue after the study had ended, and more than half of the study participants continued to attend the sessions after their participation in the study had finished.

### Quantitative findings

Table 4 summarises the quantitative findings for the study, showing the mean and SD (in brackets) for each measure at baseline and follow up. Only data from participants who completed the questionnaires at both time points are included.

**Table 4.** Summary of quantitative findings.

**Name of instrument**	**Participants with dementia**		**Carers**		**Total possible score**	**Implication of higher score**
	**Baseline**	**Follow up**	**Baseline**	**Follow up**		
**QoL-AD (/proxy)**	42.60 (4.99)	43.00 (3.70)	34.14 (7.67)	33.57 (6.92)	52	Higher QoL
**EQ-5D-5 L**	86 (16.68)	87.5 (16.05)	78.75 (16.20)	80.63 (8.21)	100	Higher QoL
**DEMQOL**	98.43 (6.29)	97.43 (7.44)			116	Higher QoL
**GDS**	1.01 (0.58)	1.74 (1.42)	1.91 (2.51)	1.75 (2.49)	15	More depressive symptoms
**MuseEQ**	111 (24.40)	112.14 (25.78)			175	More engagement with music
**IQCODE**	72.41 (10.39)	72.44 (9.61)			80	Higher cognitive decline
**BADLS**	14.89 (8.43)	14.22 (5.80)			57	More impairment with daily activities
**QCPR**			60.75 (7.98)	58.88 (8.18)	70	Higher quality of relationship
**COPE** *			a. 12.25 (2.31)	a. 11.13 (1.89)	a. 28	a. Higher negative impact of caring
			b. 13.63 (1.30)	b. 13.25 (1.67);	b. 16	b. Higher positive value of caring
			c. 9.125 (2.36)	c. 10.125 (3.09)	c. 16	c. Higher quality of support
**SSCQ**			27.75 (4.68)	27.88 (3.98)	35	Higher sense of competence

* COPE index scored in subscales; a = negative impact of caregiving, b = positive value of caregiving, c = quality of support.

Visual inspection of Table 4 indicates that for most of the measures there was very little change in the scores between baseline and follow up. This could indicate that singing group attendance did not have any effect on these domains, or it could suggest that attendance at the group meant that the participants remained stable across the course of the sessions. However, there are several points of interest in the data which are discussed below.

Firstly, participants tended to score highly on measures of quality of life (QoL-AD, EQ-5D-5L, DEMQOL), caregiving experience (COPE, SSQC) and relationship quality (QCPR), and low on the measure of depression (GDS). This indicates that despite the challenges of living with both dementia and the uncertainty of the pandemic, this group of people was managing well. Since they were not particularly depressed, had a high quality of life and were coping well with caring responsibilities, we would perhaps not expect their scores on these measures to change greatly over a relatively short space of time.

Secondly, we observed a pronounced difference between the self-rated QoL-AD scores provided by the participants with dementia, and the carers’ proxy scores. The person with dementia tends to rate their own quality of life much more highly than their carer does, around six or seven points higher on average. The finding is congruent with the paper by O’Shea et al. (2020) who report that carers rated quality of life using QoL-AD significantly lower than people with dementia. However, despite the difference in the overall scores of self and proxy ratings, people with dementia and carers seem to agree that their quality of life as measured by QoL-AD did not change greatly over the course of the study.

Thirdly, a trend which was apparent in the data related to the scores of people who were lost to follow up for various reasons. When the scores of these people were included in the analysis, the average scores were lower at baseline than at follow up for some measures, including EQ-5D-5L (for participants with dementia), QoL-AD, MUSEQ, and BADLS. The effect mostly disappeared when only complete cases were included in the analysis. This trend suggests that people who dropped out were different from people who completed the study, perhaps in that their health was less good or their dementia more severe. This observation aligns with some of the reasons that participants were lost to follow up at the end of their participation in the study.

### Qualitative Findings

Ten interviews were completed with the study participants. Three dyads did not respond to requests for interviews, and one dyad declined. Seven of these 10 interviews were with the person with dementia and carer together, and three were interviews with the carer alone because the person with dementia declined or was unable to take part. Analysis of the interview data is organised here in six categories relating to online singing: context, process, use of technology, session content, impact in the moment, and outcomes. Note that not all interview text was coded, and some text was coded at more than one theme, which is standard procedure with the chosen analytical approach. For the sake of brevity, themes relating to the participants’ experiences of the online sessions, and the effects of these sessions, are explored in more detail while other themes are summarised.

#### Category 1: Context

This category includes background data which may have influenced the experiences of the participants in the group, such as their pandemic-related life changes, motivations for attending the online group and past musical experience.

**Figure 2.**
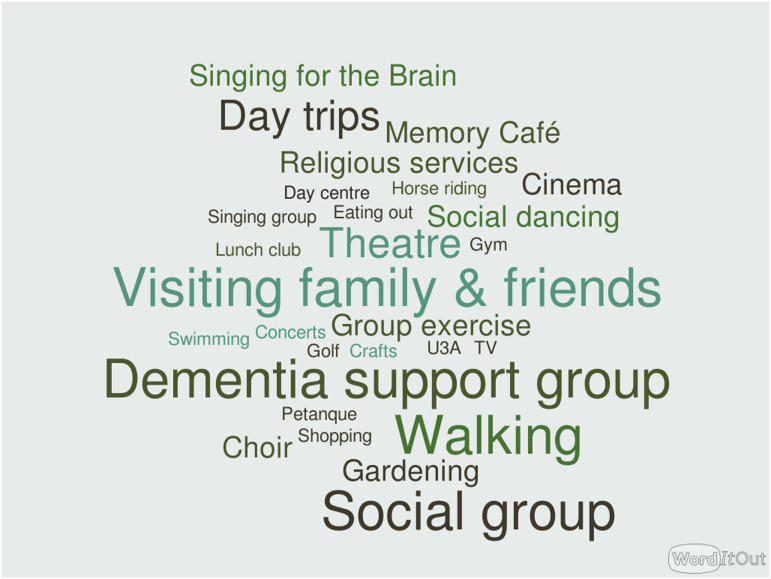
Word cloud of pre-March 2020 social and leisure activities.

**Figure 3.**
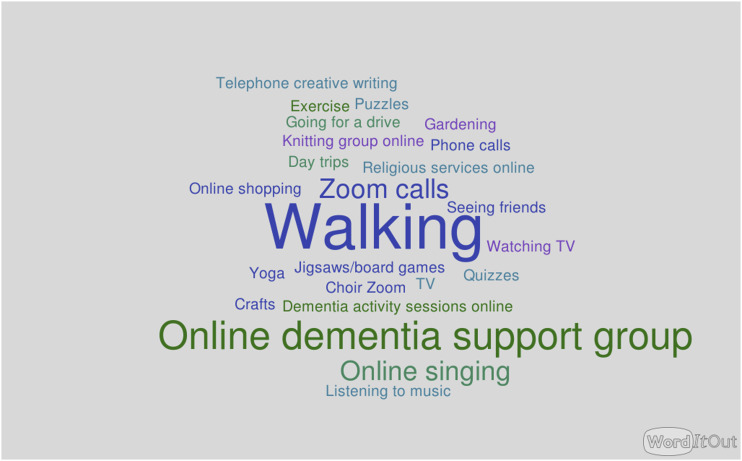
Word cloud of social and leisure activities post-March 2020.

All of the study participants discussed the effects of the pandemic upon their usual support and recreational activities, which had generally been paused completely unless they were able to move online. The word clouds (Figures 2 and 3) illustrate the activities which participants had been taking part in immediately prior to COVID-19 restrictions (before March 2020), and those they had taken part in since March 2020, up until the time they enrolled in the study. The absence of social activities and support groups from the post-lockdown opportunities is striking. No online activities figured in people’s routines pre-pandemic, but these appear in the post-COVID activities.

One carer, when asked how COVID had affected the activities they took part in, replied “There is nothing” (C04). A person living with dementia described how she “felt very upset about it really because it’s very hard to change things if you’ve got dementia, you get into a routine with things and so changing thing is really difficult, yeah?” (P05).

Motivations for attending the singing group varied. Several carers mentioned their awareness that singing could have benefits for people with dementia. For another dyad, the main motivation was that the participant with dementia was missing the singing group that she had attended in-person before the pandemic (C&P05).

All dyads reported some existing engagement with music in their daily lives, ranging from casual listening at home to involvement in singing and instrumental performance. In total, 11 participants with dementia and seven carers said that they had “quite a lot” or “extensive” prior singing experience. Three dyads had joined Singing for the Brain groups or community choirs in recent years, while four had lifelong singing experience, including one person with dementia who had been a professional singer. One of these experienced singers with dementia referred to her own sense of identity, saying “Singing, it was me” (P11).

Many participants were self-deprecating about their own musical abilities: “We like singing. We’re not very good, but we enjoy it” (C01); “I always used to say that I was the one who found the lost chord. I mean, I know I’ve got a terrible voice” (P06). A number of these participants had been told at a young age that they were “tone deaf” or unable to sing and retained this belief. Despite this, there was a sense in their comments that one did not have to excel at singing in order to find it enjoyable.

#### Category 2: Process

This category included data relating to the process of attending the groups that was not specifically about technology (see Category 3). Participants were asked to comment on how they felt the online sessions differed from face-to-face sessions. Those who had never attended a face-to-face session were invited to imagine how it might be different, while those who had previously attended singing groups had a direct point of comparison. The “Cases” feature in NViVo was used to separate those with recent experience of face-to-face singing groups from those without. It appeared that those who had recently been attending face-to-face groups were more likely to see the online group as a “better than nothing” substitute for face-to-face sessions until these were able to resume. Comments suggested that online singing was “not a patch” on face-to-face singing (P05), that it was “not the same, but at least it’s an engagement” (C09), that there was “a massive part missing” (C09), and that “there’s no comparison, really” (C11). One person with dementia commented on the sense of togetherness created through singing which was lacking in the online group: “I think it’s almost quite isolating in a way whereas when you sing in a group all the voices blend together and you know and you get used to that”. (P05) However, reasons for preferring face-to-face singing mainly related to the difficulty of socialising and interacting with other group members online, rather than the actual experience of singing itself. One carer said “Well, it’s better than nothing, but seeing people in real life and meeting up. Having a hug. Having a chat. That’s the important part.” (C08) Carers may have felt the loss of this form of peer support even more keenly than the people they care for: “Just to have a chat to other people and see how things are going, yeah, you know that that’s what has been missing for what is nearly 18 months now” (C09).

By contrast, those without recent experience of face-to-face singing groups tended to have a more mixed opinion of online singing. Although they could imagine ways in which face-to-face singing might be preferable, they also saw benefits to the online singing group and some indicated that they would prefer an online group. One person with dementia said “I think I like it like this”, although her daughter felt her mother would actually have preferred to go out (C&P01). One carer said that she had encouraged her husband to join a face-to-face singing group but he had refused. The online singing sessions seemed easier and perhaps less risky for him. Another person felt that the experience of singing while muted made people less reserved about joining in.

A number of group members commented on the fact that it was pleasant to undertake a shared activity with their care partner. One carer commented that taking part had resulted in them doing more music together outside the sessions: “It’s given us something that we could do together. Which we enjoy tremendously.” For another carer who did not live close to her father, joining the Zoom sessions gave them a different way to spend time together: “… it’s a connection with my dad, 1 hour a week in a different way […] I would never have the opportunity to take Dad to an activity or be with him during one of his activities” (C07).

#### Category 3: Use of Technology

Participants were offered technical support prior to joining the sessions, which included device-specific instructions for using Zoom, and the offer of practice Zoom calls with verbal instructions by telephone. This support was not required by any of the dyads. A researcher was present in every online singing session to help if needed, and mostly the support they provided was around muting participants or instructing them how to unmute. Only one dyad had never used Zoom prior to joining the online sessions. Among the others, either the carer or both had previous experience with using Zoom. Usually, the carer’s support was essential to manage the technology, although there were exceptions; one carer commented “Mum’s great actually with tech … Mum is very good at actually using it, you know?” meaning that she was able to use it if the initial set-up was done for her. Where care partners were not physically together when attending the sessions, solutions were devised to enable the person with dementia to take part. For example, on one occasion a person with dementia was able to join the session because their family had equipped their computer with software which allowed them to remotely take control of the computer. A carer described how a family member had changed his shifts at work so that there would be someone to assist their father with the technology needed for attending the session (C&P07).

Two dyads regularly joined the session from different locations. For one of these, the person with dementia lived alone, and before each session the care partner would phone her to talk her through the process of joining the session. The success of this approach varied; most weeks it worked, but as the carer commented “I think we missed one or two sessions at one point just because we just literally […] couldn’t get [the person with dementia] online” (C05). Although this person with dementia was generally negative about using technology, remarking “It’s a bloody nuisance honestly, technology”, the carer had noticed that her ability to use Zoom had improved during the course of the study.

Overall, there were few barriers which got in the way of participants joining the sessions but some problems were mentioned. One carer said that a couple of times the “line went dead” (C01); another found that her laptop “didn’t like Dad’s internet connection” during the first session (C06). These sometimes had simple solutions; for example, “we only had difficulty getting in once, but we went back out and in again and it worked” (C11). However, the participants who joined from their care home experienced frequent difficulties with their internet connection; the member of staff who supported them commented “the streaming was really poor and so it’s very frustrating, but we kept going in and out … I think that really put them off” (C14). Several commented on the issue of not being able to hear the session well enough. Use of multiple devices seemed to cause fluctuations in volume and maximum volume was limited by the devices participants used to join the sessions. Tablet or laptop computers and mobile phones could prove too quiet where no external amplification was used, especially if one of the participants had a hearing impairment. Aside from these technical problems, several people commented that Zoom was stable and easy to use. Some participants had no technical problems at any time. That few participants experienced technical issues apart from those mentioned above can be corroborated by the observations of the researcher who was present in all the sessions.

Many participants commented on singing while muted. Although the group understood why this was necessary, participants varied in their opinions of this experience. Some regretted they could not hear the other participants singing, to feel that they were part of a shared musical experience. One person with dementia who had plenty of singing experience commented that not being able to hear the rest of the group made him feel uncertain about whether he was in time and in tune: “Am I singing in the right key or am I keeping up with the music? Cos at the moment all I can hear is me singing” (P06). On the other hand, some participants felt more comfortable singing while they were muted, especially if they were not experienced singers: “Because it’s on mute, it’s brilliant cos you can sing away and nobody gives a monkey’s that I’m never in tune” (C09).

#### Category 4: Session Content

There was general agreement that the choice of songs used in the group was satisfactory, and that there was enough familiar material for everyone despite the age range of participants with dementia (72–92 years). About half the participants felt that the ratio of chatting, warming up and singing in the sessions was about right, while the other half would have preferred more time spent singing and thought the talking and/or warming up went on for too long, or that the session was too short overall. Two dyads commented on the lack of opportunity to have real conversations with other group members and suggested that this could be improved in future sessions.

Most participants in the sessions were from a white British background. However, one dyad was from a family with South Asian roots, and during the interview with the carer from this dyad, we discussed how cultural differences affected her father’s participation within the group (C&P07). When this dyad joined the session, the singing group leaders talked with the carer who sent them the lyrics to an Arabic song, “Ya nabi salim al’ayika” which she thought might help her father to settle into the group. The group leaders sang this song in the following session. They also added a verse including the phrase “Salaam Alaikum”, meaning “Peace be with you” to the song which the group always ended with, “Shalom”. The carer reported in the interview that her father “likes the fact that he’s integrated into it by just doing that one song, by adding Salaam Alaikum to the Shalom song”.

#### Category 5: Impact in the Moment

This category describes the short-term effects of attending the singing group sessions as described by the participants, including positive, negative and neutral aspects, occurring during the session itself. The longer-lasting effects of the sessions are described later, in “Outcomes”. We found that there were six themes relating to “impact in the moment”, each captured by a verb: enjoy, occupy, interact, transform, think, observe.

##### Enjoy

All the interviewees made reference to the sessions being enjoyable, either by explicitly stating this, or by saying that singing brought them pleasure. Comments included “We like singing. We’re not very good, but we enjoyed it” (C01); “it’s good fun” (P04); “I love singing. I really love singing.” (P05); “I just get a lot of satisfaction out of it” (P06); “We enjoy the singing and the chatting, I think.” (C08).

##### Occupy

Attending the singing group was seen by many of the participants as a way of passing the time, during a period in their lives when most of their usual activities had stopped completely. One carer commented that her motivation for getting involved was “obviously with COVID just to give mum something to do and for us both to do something that we enjoy” (C01). A person with dementia described how the group helped to keep her busy: “Yes, obviously it does have some benefits because you know, it fills in some other part of a day. You know? It can be very long and. And I’m very extroverted so I can’t sit at home being bored for very long” (P05).

##### Interact

Several interviewees commented that, despite the limitations of online singing, they appreciated the opportunity to meet and interact with other people through the group sessions. One carer commented that her father “looks forward to it and likes the interaction” (C07). Another carer said an impact of the session was that “you don’t feel so isolated”. The sessions were a rare opportunity to meet new people during a time of lockdown: “I think it’s been nice to have an interaction with different people. […] Especially when we’re chatting.. yes, it has had an impact in that respect” (C09). While for some respondents the online sessions were able to recreate something of the experience of being in a group with other people, others indicated that interaction was impeded by the medium: “And I think it’s actually hearing other people singing, then feeling you’re joining in more, when you’re actually present” (C11).

##### Uplift

Five dyads mentioned positive, short-term, transformational effects on their mood or wellbeing. One person with dementia commented “you enter a different world when you’re singing I think” (P05). Several people described the effect that the sessions could have on their mood, often using the word “uplifting” to capture the essence of the experience. Another person described how singing made him feel happier: “I enjoy singing, it keeps me happy. And while I’m in the singing group, I’ve found that it's bringing my happy, happy moods out to the forefront more than they were before” (P06). These impacts were evident to carers: “It can be a mood changer from the beginning of the session. Can both come with long faces, having had perhaps difficult morning or we’re tired. And then by the end of the session we can be jolly and singing. We can be together. And it’s wonderful” (C11).

##### Stimulate

Four interviews contained comments about how the sessions stimulated thinking, learning and remembering among the people with dementia and their carers. One dyad spoke in their interview about how the sessions had made them think more about the meanings behind the words of the songs. Two dyads described how familiar songs from the past had stimulated the memory of the person with dementia. One carer commented that her father enjoyed the old songs from musicals and films he remembered watching on the television as a family, even though at the time he had not been particularly into the music itself (C07). She felt that the combination of making connections with other people in the group and the reminiscence from singing was “re-energising Dad’s brain”. The member of staff who supported residents from the care home talked about how the combination of remembering the songs and using other skills at the same time (such as turning the pages of a songbook) was both stimulating for the residents and useful for her to see how they coped with these tasks (C14).

##### Compare

Several carers also mentioned the opportunity afforded by the sessions to observe the responses of people with dementia. There were two different facets to these observations. For the carers who were joining the sessions while physically apart from the person they cared for, the group offered a chance to check in and see how they were doing. There were also comments about how the sessions provided the opportunity to see how the responses of the person they cared for compared to the other people with dementia in the session. One carer appreciated the chance to “see other people involved and their carers and things and see how people are doing generally” (C05). Another carer was able to compare her mother’s responses to those of other people with dementia: “I’m interested in the behaviour of the other people that have got signs of dementia or something that to effect really and see how they’re interacting and how mum’s interacting” (C11).

#### Category 6: Outcomes

This category describes more long-term effects which participants attributed to attending the group sessions. Three carers remarked on the fact that they could see observable benefits in the person they cared for. For instance, one commented “I know that it's beneficial ‘cause you can see the change” (C09) while another, who was a healthcare professional, commented “I know the theory of it as a nurse, but to see it for real and see that growth in Dad, you know, I just say it is amazing” (C07). More specifically, the outcomes noted fell into several themes: effects on daily life, musical activities, wellbeing, and social interaction.

##### Effects on Daily Life

In half of the interviews, participants said that coming to the sessions gave them something to look forward to. Some mentioned this several times, emphasising its importance. A typical comment from a person with dementia was “I found it very helpful and I’ve enjoyed it and it’s something I look forward to” (P11). Relatedly, half of the interview participants said that coming to the group provided them with a sense of routine. This was felt to be especially important in light of the disruption caused by lockdown restrictions: “You know it’s because routine and what you’re doing on a certain day in these circumstances is important” (C11).

##### Engagement With Music in Life

Most of the participants made some comment on whether and how the sessions had affected the presence of music in their lives. Four dyads expressed an intention to continue attending the singing group sessions after their participation in the study had ended. Another four said that they would think about seeking out a face-to-face singing group when this became possible, although the carer from one of these dyads expressed reservations about whether her husband would actually want to go to a face-to-face group (C13).

Some people described ways in which the group had affected their engagement with music outside the sessions. One carer said that she and her father used the songbooks from the sessions to sing along to YouTube videos, with her father commenting, “We have a good time. It’s very enlightening” (P06). The same person also said that coming to the group had changed how he felt about his musical abilities, commenting “I’m improved in my singing”.

##### Wellbeing

A number of comments referred to the effect that the sessions had on participants’ general sense of wellbeing. Four interviews made reference to lasting improvements in mood; one person with dementia said: “I think I feel somewhat happier. I’m humming the songs that we’ve been singing with the group. I’ve still got me book. And if I’m in a happy mood I’ll get it take it, take it up and start singing some of the songs you know?” (P06) In the case of this person, it seemed that not only did the sessions improve his mood, but the carryover from the sessions into his everyday life gave him a tool to improve his wellbeing during the week. One carer said that, although her care partner did not remember having done the sessions shortly after they had finished,: “I think I have noticed there’s certainly wellbeing impact, I would say probably the rest of the day” (C13).

Also relevant to wellbeing were comments indicating that some people with dementia found the experience of attending the sessions quite tiring. One carer said that “A couple of times mum’s gone straight to bed afterwards” and described how she found the first few sessions very tiring, but that her stamina had improved once she had got used to it (C04).

##### Relationships

Three dyads felt that the sessions had had some positive effect on their relationship. One carer thought that simply having an activity which they could do together again was beneficial, and her mother agreed (C&P01). Another carer described how the sessions could dissolve tension between him and his wife: “It can be a mood changer if there’s a little bit of angst between us. At the start, because I am losing patience, and an hour on the session, and it’s dissipated. It’s gone, you know it can be holding hands with singing together. Smiling.” (C08) Another carer simply said “It’s like for that hour […] I’ve got my dad back.” (C07) Her words imply that the effect of the music helped them to reconnect. Three other dyads said that they felt the sessions had not had an effect on the relationship between them.

There were not many comments about getting to know people through the online sessions, perhaps because of the previously-discussed difficulties of interacting on Zoom. One person with dementia said that “meeting people with similar interests” was a positive outcome of the sessions, and that it was “very nice to meet other people or talk to other people. Sing together.” (P04) Another carer commented that it was good to know something about the other group members, and that as time went on it felt like you were getting to know them: “I think gradually over the weeks you do get you get more and more, don’t you, you get little titbits every time rather than getting a whole lot about a person right at the outset” (C11).

## Discussion

Overall, the response to the online singing group was mostly positive. Participants were able to join the sessions using the required technology and had few technical issues in doing so. Although the limitations of singing online were noted by the participants, the sessions were frequently described as enjoyable and had both short- and longer-term benefits for many of those who attended. Some people saw advantages to the online format such as improved accessibility and reduced self-consciousness. However, those who had previously attended face-to-face singing sessions felt that the online sessions were “better than nothing” but did not compare favourably to live sessions.

The people who took part in this study had a range of different experiences of singing and music. Some were accomplished life-long singers and musicians; others believed they were unable to sing or were tone deaf. All these people took part together but, unlike in a face-to-face singing group, they were unable to hear each other. This unique feature of online singing groups could be empowering for people who felt shy or self-conscious about singing in front of others, but might be sub-optimal for those with singing expertise as they do not get the opportunity to have their skill or any improvements acknowledged. Their voices remain unheard.

Online activities were clearly needed because of the pandemic. One carer related lockdown to worsening of her father’s dementia, and there is already some research evidence to support the hypothesis that the pandemic accelerated cognitive decline among people with dementia (Ismail et al., 2021). Study participants with previous experience in singing groups were prepared to accept online singing as a substitute but would not have chosen it if face-to-face singing had been available. Others who had only attended online singing were more likely to see some advantages, and it could potentially provide a gateway to face-to-face singing. For example, being muted while singing seemed to give some people the extra confidence they needed to join and participate in the session. We acknowledge that online activities are not accessible to everyone, and clearly a range of alternatives are needed to avoid excluding those without access to the internet. For instance, landline telephone conferencing has been used to host singing groups for people with dementia during the pandemic; this also has the advantage of reduced audio latency (Edwards, 2022).

The dynamic between the person with dementia and their carer is perhaps even more important for online singing groups than it would be in-person. Most of these people were living together, and the research was conducted at a time when people were confined to their homes, so it is interesting that, despite spending this time together, there was an appetite for more structured activities involving co-participation. Several carers reported looking to other dyads to compare how the person they cared for was interacting and to find affirmation in their own decisions about what level of support to provide. The generally positive experiences of care partners who joined the sessions separately from different locations suggest that online singing groups may provide a way for people with dementia to participate in group activities with geographically remote family members and friends, an advantage which will remain relevant beyond the pandemic. For both co-habiting and remote care partners, having something structured to do together proved important.

However, the importance of the group facilitators should not be underestimated. Whichever the mode of delivery, they are responsible for building and maintaining the sense of group feeling. Arguably, this is much harder for an online group, where no one can hear the whole group singing, and participants’ ability to interact with each other is more limited. The facilitators in this study helped to create this sense of group feeling by creating a predictable structure for the sessions (e.g. by starting and ending with same activity/songs each week), facilitating dialogue which allowed people to get to know each other, acknowledging landmarks like birthdays in the group, and by encouraging contributions from the group members (e.g. making space if someone wanted to sing a solo or say something to another group member).

There were very few persistent technological problems during the course of the study, the exception being the care home which struggled with the reliability of its internet connection. It was reported in 2020 that according to data from the Care Quality Commission, 7000 care homes in England were without an adequate internet connection, so this is clearly not a unique problem, although the pandemic has undoubtedly changed the situation (Thorn, 2020). The most commonly-reported disadvantage of online sessions was the difficulty in interacting with other people. This was seen as a major drawback, especially by people who knew what in person singing groups were like. We know from existing research that this is a very important aspect of face-to-face singing groups, and that carers greatly value the opportunity for peer support and resource sharing (Osman et al., 2014). We also note that some level of knowledge about technology was necessary for taking part in this study, and observe that 50% of participating carers were the adult children of the person with dementia. It seems possible that people with dementia who had a carer from a younger generation were more likely to take part in this study as they were more likely to already possess the technical skills.

Our data analysis separates “in the moment” impacts from longer term outcomes. The idea is that benefits which occur during the group may translate into longer-term ones if they are adequately sustained. The mechanisms which explain these translations are easy to describe in some cases. For example, having more opportunity to interact with others on a regular basis (impact in the moment) may lead to someone feeling less isolated (outcome). More work is needed to demonstrate causal inks, but if we want to explore with further research the possibility of longer-term benefits from attendance at group singing, the findings here suggest that wellbeing, routine and enjoyment, social connection and support, and increasing the availability of musical resources in daily life, are what matters to people who attend group singing sessions, whether online or in person.

**Figure 4.**
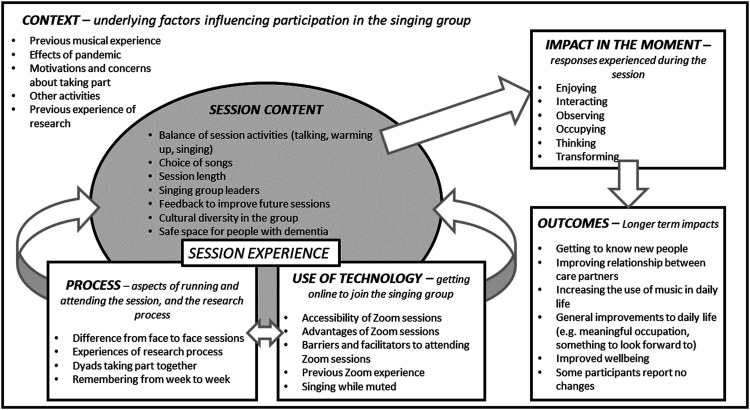
Diagram depicting qualitative themes and the relationships between them.

Figure 4 visually represents the categories and themes which were identified during the analysis, and proposes a relationship between them. The underlying Context is considered to potentially influence all aspects of participation in the singing group, since factors such as participants’ previous experience, motivations, and the effects of the pandemic will affect both engagement in the group and any short- or long-term effects. The Session Content, Process and Use of Technology categories are closely related, and together they have been termed “Session Experience”. The participants’ overall experience in the session will affect how the session impact them “in the moment”, which in turn may contribute to developing and sustaining longer term outcomes. What the diagram does not capture is the regular, routine nature of the sessions which may help any longer-term benefits to coalesce and be maintained over time.

### Limitations

This study reports the experiences of a small group of people who were opportunistically selected. We cannot be sure that their experiences are more broadly representative of people with dementia and their carers. However, singing is a popular activity and many other online singing groups sprang up during the pandemic, so the experiences of our study participants are probably fairly similar to other members of this population who had the resources and skills to access online singing. Although we were prepared to provide technical support, we were not able to provide devices and internet connections for those without, so people who did not already have the necessary technology were excluded from taking part. Since the study was advertised mainly online, and participating involved interaction with technology, it is likely that the sample was skewed towards people who had pre-existing technical competence. This may mean that participants were more likely, for example, to be younger or of a higher socio-economic status than the general population. The interviews were conducted after the 10 sessions and therefore may have been affected by subjective recall bias.

## Conclusion

This proof-of-concept study of online singing has demonstrated that it possible for a group of people with dementia and their carers to attend singing sessions using videoconferencing. Benefits during the group and ongoing benefits were widely reported, and most found these sufficiently engaging to continue attending after the study ended. Our research also showed that it is possible to conduct data collection online with people with dementia and their carers, using face-to-face video interviews and online forms.

The pandemic has brought with it many creative responses to challenging situations and has tested the resilience of people with dementia and those who care and provide services for them. Online sessions have various potential advantages: they may offer a lower-pressure environment for people to try out singing, they allow family and friends to attend sessions with geographically distant relatives, and they are more accessible for the house-bound. Although these groups evolved in response to a specific situation, they may have implications for future practice. As singing groups start to resume in person, we may reflect on what the online singing experience can offer beyond the pandemic, and whether there will be a demand for online singing alongside in-person sessions. In particular, future research in this area could productively explore how the musical experience of online singing can be optimised, how the social features of group singing can be translated into an online format, and how online activities and services for people living with dementia can successfully balance accessibility needs on one hand with the risk of digital exclusion on the other.

## Supplemental Material

Supplemental Material - Online Singing Groups for People With Dementia: Adaptation and Resilience in the Face of the COVID-19 Pandemic Supplemental Material for Online Singing Groups for People With Dementia: Adaptation and Resilience in the Face of the COVID-19 Pandemic by Becky Dowson, Justine Schneidera, Orii McDermotta, and Martin Orrell in Dementia
