# Supplementary material for: Online Singing Groups for People With Dementia: Adaptation and Resilience in the Face of the COVID-19 Pandemic
Source: Dementia (London). 2023 Jun 9;22(7):1348–71. doi: 10.1177/14713012231179262 (PMC10261964; doi:10.1177/14713012231179262)
Supplement: Supplemental Material - Online Singing Groups for People With Dementia: Adaptation and Resilience in the Face of the COVID-19 Pandemic [file sj-pdf-1-dem-10.1177_14713012231179262.pdf]

**Supplementary Material: List of songs included in songbook created for the study**

| | |
|---|---|
| Annie‘s Song | My Favourite Things |
| Black Hills Of Dakota | Oh What A Beautiful Morning |
| Bobby’s Girl | Que Sera Sera |
| Bring Me Sunshine | Rock Around The Clock |
| Bye Bye Love | Runaway Train |
| Catch A Falling Star | Save All Your Kisses For Me |
| Clementine | Secret Love |
| Cockles And Mussels | She Loves You |
| Country Roads | Skye Boat Song |
| Dancing Queen | Slow Boat To China |
| Delilah | Somewhere Over The Rainbow |
| Downtown | Summer Holiday |
| Edelweiss | Supercalifragilistic |
| Fly Me To The Moon | Swinging On A Star |
| Get Me To The Church | That’s Amore |
| Getting To Know You | These Boots Are Made For Walking |
| I Can See Clearly Now | Those Were The Days |
| I Do Like To Be Beside The Seaside | Tie A Yellow Ribbon |
| I’d Like To Teach The World To Sing | Top Of The World |
| Kiss Me Honey Honey | Tulips From Amsterdam |
| Let's Go Fly A Kite | Walking Back To Happiness |
| Morning Has Broken | Windmill In Old Amsterdam |
| Morning Town Ride | Wonderful World |
| Music Music Music | Yellow Submarine |
| | Shalom |
